# Angiopoietin-2 Primes Infection-Induced Preterm Delivery

**DOI:** 10.1371/journal.pone.0086523

**Published:** 2014-01-21

**Authors:** Electra N. Polyzou, Nikolaos E. Evangelinakis, Aikaterini Pistiki, Antigone Kotsaki, Charalampos S. Siristatidis, Charalambos G. Chrelias, Emmanuel Salamalekis, Demetrios P. Kassanos, Evangelos J. Giamarellos-Bourboulis

**Affiliations:** 1 3^rd^ Department of Obstetrics and Gynecology, University of Athens, Medical School, Athens, Greece; 2 4^th^ Department of Internal Medicine, University of Athens, Medical School, Athens, Greece; Xavier Bichat Medical School, INSERM-CNRS - Université Paris Diderot, France

## Abstract

Current knowledge on the participation of angiopoietin-2 (Ang-2) in the inflammatory process and on the importance of bacterial endotoxins (LPS) in the induction of preterm delivery (PTD) led us to investigate the role of Ang-2/LPS interplay in the pathogenesis of PTD. At a first stage, Ang-2 was measured at the end of the first trimester of pregnancy in the serum of 50 women who delivered prematurely; of 88 women well-matched for age and parity who delivered full-term; and of 20 non-pregnant healthy women. Ang-2 was greater in pregnant than in non-pregnant women. The time until delivery was shorter among those with Ang-2 greater than 4 ng/ml (odds ratio for delivery until week 34; p: 0.040). To further investigate the role of Ang-2 for PTD, an experimental model of PTD induced by the intraperitoneal injection of LPS in mice was used. Ang-2 was administered intraperitoneally before LPS on day 14 of pregnancy. When Ang-2 was administered before the LPS diluent, all mice delivered full-term. However, administration of Ang-2 prior LPS accelerated further the time until delivery. Sacrifice experiments showed that the effect of Ang-2 was accompanied by decrease of the penetration of Evans Blue in the embryos and by increase of its penetration in maternal tissues. In parallel, the concentration of tumour necrosis factor-alpha in the maternal circulation, in fetal tissues and in the placentas was significantly decreased. Results indicate that Ang-2 accelerated the phenomena of PTD induced by LPS. This is related with deprivation of fetal perfusion.

## Introduction

Preterm labor and delivery (PTD) is defined as childbirth occurring between the 20^th^ and 37^th^ week of gestation [1], [2]. PTD remains the most important unsolved problem in obstetrics. Despite existing interventions like the use of tocolytics and antibiotics, the incidence of PTD in the United States has not decreased during the two last decades and remains at the levels of 12% of pregnancies [3]. Although the etiology is multifactorial, approximately 30–40% of PTD are associated with an underlying infectious process [3].

Angiopoietins (Angs) are protein growth factors that promote angiogenesis. There are four identified angiopoietins (Ang-1, Ang-2, Ang-3, Ang-4). Ang-1 is required for the formation of mature blood vessels whereas Ang-2 promotes vascular leakage [4]. Both participate in systemic inflammation [5]; as a consequence they are expected to participate also in the process of PTD stimulated by an intrauterine infection.

Two recent studies of our group have shown that Ang-2 possesses considerable anti-inflammatory properties. More precisely, pre-treatment of mice with Ang-2 is accompanied by prolongation of survival after infection by multidrug-resistant *Pseudomonas aeruginosa* [6]. The protective effect is mediated by inhibition of the production of TNFα through an antagonism with bacterial endotoxins for TLR4 (Toll-like receptor-4) [7]. These findings lead to the assumption that Ang-2 may play a significant role in the process of PTD stimulated by bacterial infection.

To investigate the probable role of Ang-2 in PTD, we designed a two-step approach: i) circulating Ang-2 was measured at the end of the first trimester of pregnancy in a cohort of women who delivered prematurely; and b) a model of PTD after injection of bacterial endotoxin was studied. The use of this two-step approach was done on the hypothesis that elevated circulating Ang-2 was expected early among women who delivered prematurely. Then pre-treatment of an animal model with PTD with Ang-2 would allow to repeat this setting in animals and to confirm a role of Ang-2 in the pathogenesis of PTD.

## Patients and Methods

### Study Cohort

A prospective study was conducted in pregnant women under follow-up at the 3^rd^ Department of Obstetrics and Gynecology of ATTIKON University Hospital between March 2008 and July 2011. The study was approved by the Ethics Committee of ATTIKON University Hospital. Written informed consent was given by participants. Blood was sampled from these women during the 12^th^ week of pregnancy when they were screened for prenatal abnormalities after venipuncture of one forearm vein under sterile conditions. Blood was collected into sterile and pyrogen-free tubes and centrifuged. Serum was kept refrigerated at −80° until processing. Once samples were collected, it was selected which of these samples should be used for measuring concentrations of circulating Ang-2. Information for the week until delivery was available for all screened women. It was decided that all women with PTD should be included; women delivering at full-term, well-matched for age and parity should be included as comparators. Exclusion criteria for the PTD and the comparator group were: a) infection by HBV (hepatitis B virus), HCV (hepatitis C virus) and HIV (human immunodeficiency virus); b) diagnosis of eclampsia or pre-eclampsia; and c) infection by CMV (cytomegalovirus) and *Toxoplasma gondii*.

The study hypothesis was that circulating Ang-2 would be greater in 20% of women delivering prematurely compared to controls. Fifty women delivered prematurely without having any of the above exclusion criteria; to achieve the study hypothesis at power of 80% and at the significance level of 10% 88 pregnant women well-matched for age and parity should be enrolled. In order to select the 88 comparators, all women delivering full-term were classified by chronological order of the time of blood sampling and divided into sets of 10. The second woman of each set was selected. Women matched for age and parity from these selections, were used to form the comparator group. For comparisons, blood was also collected from 20 non-pregnant healthy women of reproductive age.

Ang-2 was measured in serum in duplicate by an enzyme immunosorbent assay (R&D Inc, Minneapolis, USA). The lower limit of detection was 15 pg/ml.

### Animal Study

The study received permission from the Ethics Committee for Experimental Surgery of the Veterinary Directorate of the Perfecture of Athens according to the Greek legislation in accordance with the 160/1991 Council Directive of the EU (license number: K/7167/27-10-2008). To minimize animal suffering all experiments were run under light ether anesthesia whereas paracetamol suppositories were administered every 12 hours for the first 48 hours after intraperitoneal injections.

Eight to ten-weeks old C57BL/6 male and female mice were purchased from Alexander Fleming Institute (Vari, Greece); they were allowed to acclimate for 72 hours before beginning the experiments. After acclimatization, mice were kept at cages with constant rotation rate of 70 air-changes per hour to ensure sterility (IVC SYSTEM, Technicplast, Buguggiate, Italy). Mice were fed standard chow (type 4rf 18) and were allowed water *ad libitum*. Room temperature ranged between 18 and 22°C; relative humidity was between 55% and 65% and the light/dark cycle was 6 a.m.–6 p.m.

A model of PTD was studied as previously described [8]. Briefly, after overnight cohabitation using a single pair of mice per cage, females with vaginal plugs were segregated in individual cages, weighed daily and given food and water *ad libitum*. Gestational age was determined by the presence of vaginal plug and designated as day 0 of pregnancy. The females that were not pregnant after cohabitation were not used again for this experiment. On day 14.5 after plugging (approx. 75% of the typical 19–21 day gestation), animals were anesthetized with metofane [methoxyflurane 2,2-dichloro-1,1 difluoroethyl methyl ether in butylated hydroxytoluene (0.01% w/w)] and intraperitoneally (i.p.) injected with purified lipopolysaccharide of *Escherichia coli* serotype 055:B5 (Sigma Chemical Co., St. Louis, MO) dissolved in water for injection (Bioser SA, Athens, Greece) at a concentration of 1 mg/ml. The solution was diluted into saline and kept at −4°C. The mice were lighted anesthetized by ether so that injury of the bowel or the uterus was avoided during the i.p. injection. In the series of experiments, PTD was defined as the finding of at least one fetus in the cage or in the lower vagina within 24 h of challenge with LPS. Vaginal bleeding alone was not considered evidence of delivery. Following LPS, the dams showed no evidence of serious disease. Pregnant mice were randomly assigned into the following groups where all interventions were done under light ether anesthesia:

- Group A (controls, n = 16), these mice were injected ip 100 µl of WFI on the day 14.5 after plugging. This was followed by the ip injection of another 100 µl of WFI two hours later.
- Group B (LPS, n = 16), these mice were injected ip 100 µl of WFI on the day 14.5 after plugging. This was followed by the ip injection of 2 mg/kg of LPS at a volume of 100 µl two hours later.
- Group C (Ang-2, n = 16), these mice were injected ip 3.75 µg/mouse of human recombinant Ang-2 (R&D Inc., Minneapolis, USA) diluted into WFI at a volume of 100 µl of WFI on the day 14.5 after plugging. The dose of Ang-2 was selected in analogy to one former study of our group [6]. This was followed by the ip injection of WFI at a volume of 100 µl two hours later.
- Group D (Ang-2+LPS, n = 20), these mice were injected both Ang-2 and LPS at the doses and concentrations described above.

In six female mice of each group, intense follow-up was done every two hours to record all deliveries for a total of 168 hours after the injection of LPS. The remaining mice were sacrificed at 12 hours after the injection of LPS. More precisely, animals were anesthetized with metofane when signs of labor appeared, like dilatation of cervix or vaginal bleeding (10–12 h after the injection of LPS). Through a 2 cm upper midline abdominal incision, the peritoneal cavity was entered and the intestines were displaced to the left, so that post canal vein was revealed. Evans Blue leakage into tissues was assessed. More precisely, the abdominal aorta was punctured under aseptic conditions and three ml of Evans Blue (AlterChem Co, Athens, Greece) was injected into the abdominal aorta within 5 minutes. Ten minutes after the end of the infusion of Evans Blue, repeated intravenous infusions of 0.9% sodium chloride were performed followed by animal sacrifice; the right kidney and the right lung of the mice were removed and the same was done for all fetuses and for all placentas. To minimize suffering, mice were euthanized by one 5 mg/kg intramuscular injection of ketamine.

Tissues were homogenized and centrifuged. The optical density of the supernatants was visualized at 450 nm. In another series of experiments, following animal sacrifice, blood was collected from the abdominal aorta of pregnant mice and collected into sterile and pyrogen-free tubes. Blood was centrifuged and the supernatant was collected and stored at −70°C until assayed. In the same experiments, all fetuses and placentas were removed and weighted. One ml of sterile 0.9% sodium chloride was added and tissues were homogenized with a grinder. Then the homogenate was centrifuged and stored at −70°C until assayed. Concentrations of tumour necrosis factor-alpha (TNFα) were measured in serum samples and in supernatants in duplicate by an enzyme immunosorbent assay (R&D). The lower limit of detection was 15 pg/ml. Results for tissues were expressed as pg/g of tissue after adjustments for tissue weight.

### Statistical Analysis

Quantitative demographic characteristics of women were expressed as means ± SD; comparisons were done by the Student’s “t-test”. Serum Ang-2 was expressed as medians and 95% confidence intervals (CI). Comparisons of serum Ang-2 between groups were done by the Kruskal Wallis test with adjustments for multiple comparisons by Bonferroni. Receiver Operator Characteristics (ROC) curve analysis was done to define a concentration of Ang-2 with negative predictive value more than 80% to predict PTD until week 34. Odds ratio (OR) and 95%CI of this cut-off were determined by Mantel and Haenzel’s statistics. The time of delivery after injection of LPS in mice was determined by Kaplan-Meier analysis; comparisons between groups were done by the log-rank test. Concentrations of Evans blue and of TNFα were expressed by their mean ± SE. Comparisons between groups were done by ANOVA with *post-hoc* Bonferroni corrections. Any value of below 0.05 was considered significant.

## Results

**Figure 1:**
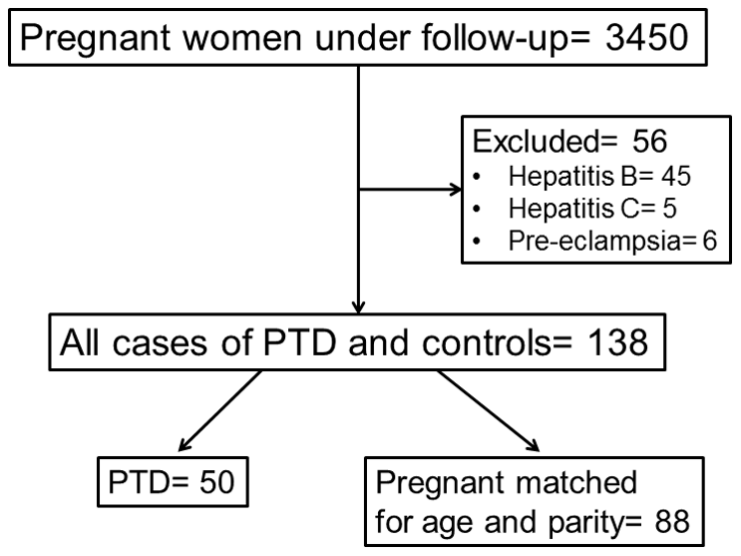
Study flow-chart. PTD: preterm delivery.

The study flow-chart is shown in Figure 1. From a total of 3450 screened pregnant women, 138 were analyzed; 50 delivering prematurely and 88 delivering at full-term. Mean ± SD age of women who delivered prematurely was 32.2±2.9 years and of those delivering full-tem 31.9±3.7 years (p: 0.794). Mean ± SD parity was 1.5±0.7 and 1.6±0.7 (p: 0.512) respectively. Mean ± SD age of the 20 studied non-pregnant healthy women was 30.9±4.9 years. None of women who delivered prematurely was diagnosed with some underlying infection.

**Figure 2:**
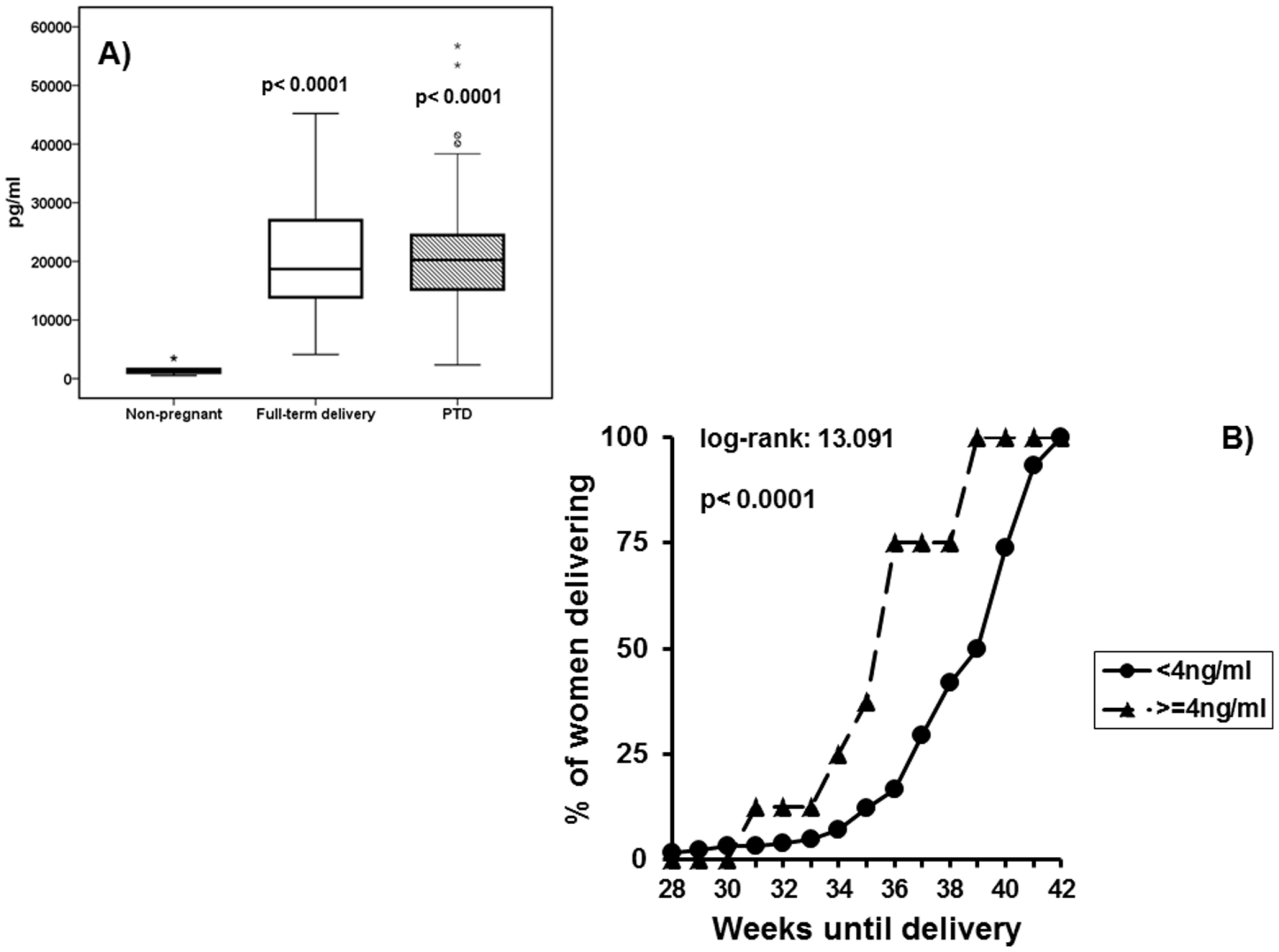
Concentrations of Ang-2 during pregnancy. A) Serum levels of Ang-2 were measured at week 12 in serum of 50 women who delivered prematurely (PTD); of 88 women who delivered full-term; and of 20 non-pregnant healthy women. P values represent comparisons to non-pregnant healthy women. B) Time until delivery in relation with the level of circulating Ang-2 at week 12. P is the value of comparisons between women with serum Ang-2 below and above 4 ng/ml.

Circulating Ang-2 in serum of pregnant women was greater than in non-pregnant women (Figure 2A). No differences were found between women who delivered prematurely and women who delivered full-term. ROC analysis identified that serum Ang-2 more than or equal to 4 ng/ml had negative predictive value 82.2% to predict PTD. Women with such high-levels of Ang-2 delivered earlier than women with lower levels of Ang-2 (Figure 2B). OR for PTD as early as week 34 with Ang-2 more than or equal to 4 ng/ml was 4.61 (95%CI: 1.07–19.79; p: 0.040).

**Figure 3:**
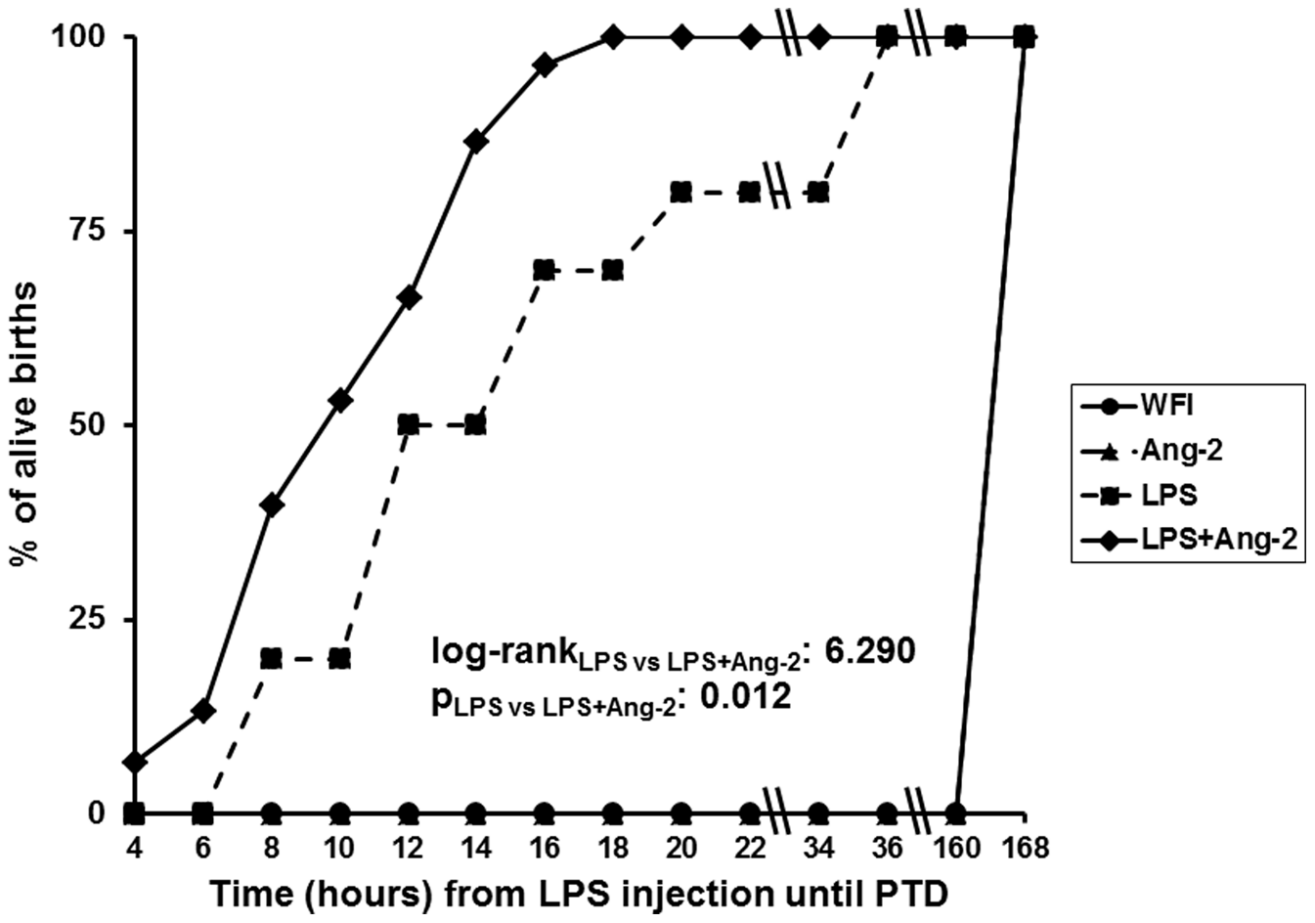
Ang-2 accelerates preterm delivery (PTD). Female pregnant female mice were challenged at 14.5 days after plugging with water for injection (WFI, group A, n = 6), with recombinant human angiopoieting-2 (Ang-2 B, n = 6), with lipopolysaccharide of *Escherichia coli* O55:B5 (LPS, n = 6) and with Ang-2 followed after two hours with LPS (Ang-2+LPS, n = 6). The time until delivery of alive offspring was recorded every two hours after challenge with LPS. Statistical comparisons between groups after correction for multiple testing by Bonferroni were: log-rank_WFI vs LPS:_ 13.679, p: 0.000217; log-rank_WFI vs LPS+Ang-2_: 15.251, p: 0.000094; log-rank_LPS vs LPS+Ang-2_: 6.290, p: 0.012.

These findings prompted us to investigate how elevated Ang-2 may contribute to PTD. To this end, we studied the hypothesis what the impact of the injection of Ang-2 may be in a model of PTD stimulated by LPS. The injection of Ang-2 2 hours before the injection of LPS accelerated preterm delivery compared to the injection of only LPS (p: 0.012, Figure 3). On the contrary, the injection of Ang-2 2 hours before the injection of WFI (group B) did not alter the time until delivery in relation to the injection of WFI (group A) i.e. the diluent of Ang-2 (p: 1.000 group A versus group B).

**Figure 4:**
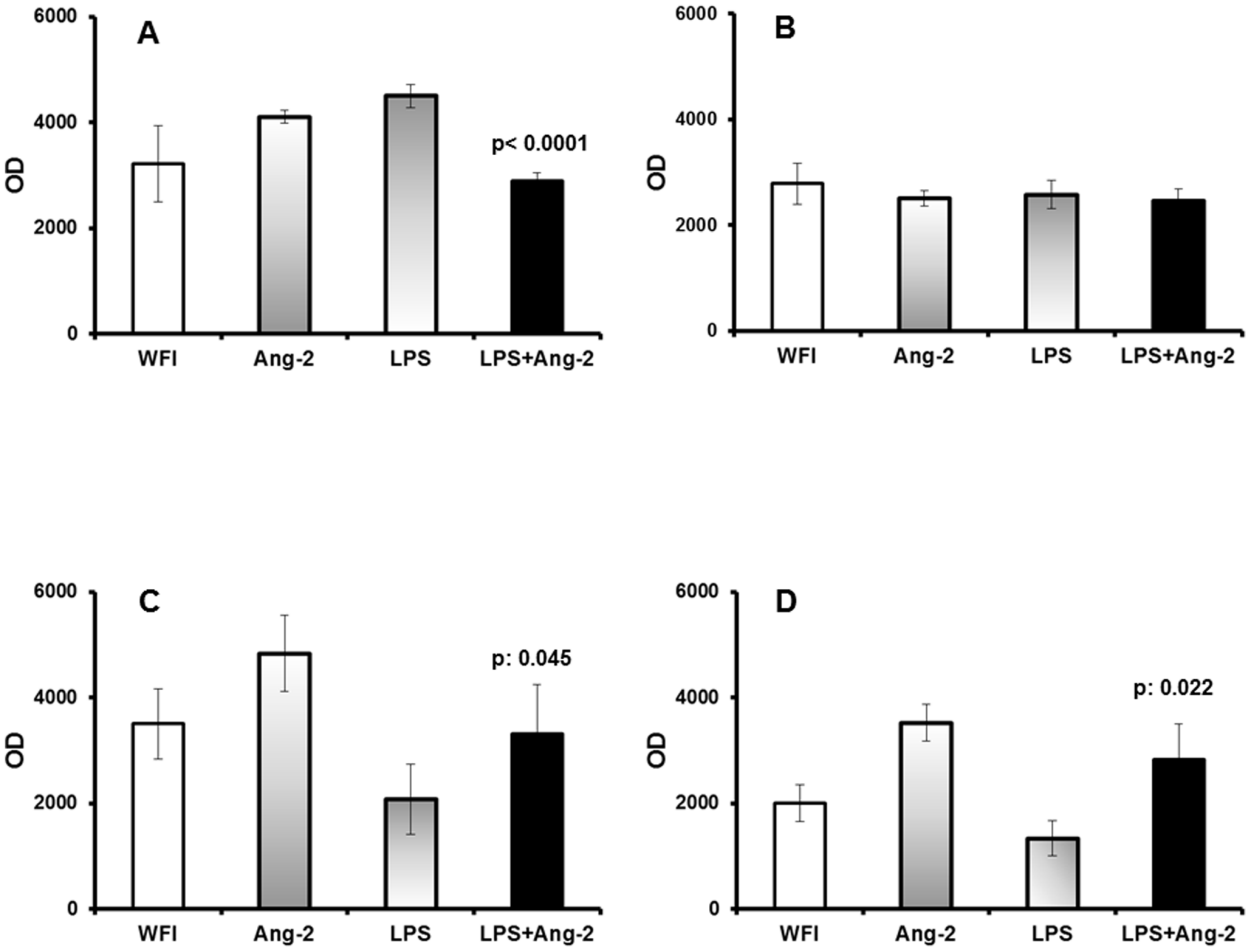
Tissue penetration of Evans blue. Female pregnant female mice were challenged at 14.5 days after plugging with water for injection (WFI, group A, n = 5), with recombinant human angiopoietin-2 (Ang-2 B, n = 5), with lipopolysaccharide of *Escherichia coli* O55:B5 (LPS, n = 5) and with Ang-2 followed after two hours with LPS (Ang-2+LPS, n = 7). One day after challenge with LPS, mice were sacrificed. Evans blue was measured in the fetuses (panel A), in the placentas (panel B), in the kidneys of mothers (panel C) and in the lungs of mothers (panel D). P values indicate statistical significances between the LPS and the LPS+Ang-2 groups.

Based on these results, we hypothesized that Ang-2 may accelerate PTD induced by LPS by one of two mechanisms: either by an effect on vascular permeability or by an effect on the LPS/TNFα interplay. The hypothesis for an effect on the LPS/TNFα interplay was based on previous findings of our group in models of sepsis [7], [9]. In order to investigate the effect on vascular permeability, concentrations of Evans blue were measured in the fetuses, in their placenta and in the organs of the mothers. Results indicated that the concentration of Evans blue in the fetuses of group D was significantly lower than in group C (p<0.0001, Figure 4A). However, the placental concentrations did not differ between groups (Figure 4B). On the contrary, the concentration of Evans blue in the kidneys and in the lungs of mothers of group D was higher than in group C (Figures 4C and 4D). These findings indicate changes in vascular permeability as part of the mechanism of action of Ang-2 towards lower perfusion of fetuses of group D than group C.

**Figure 5:**
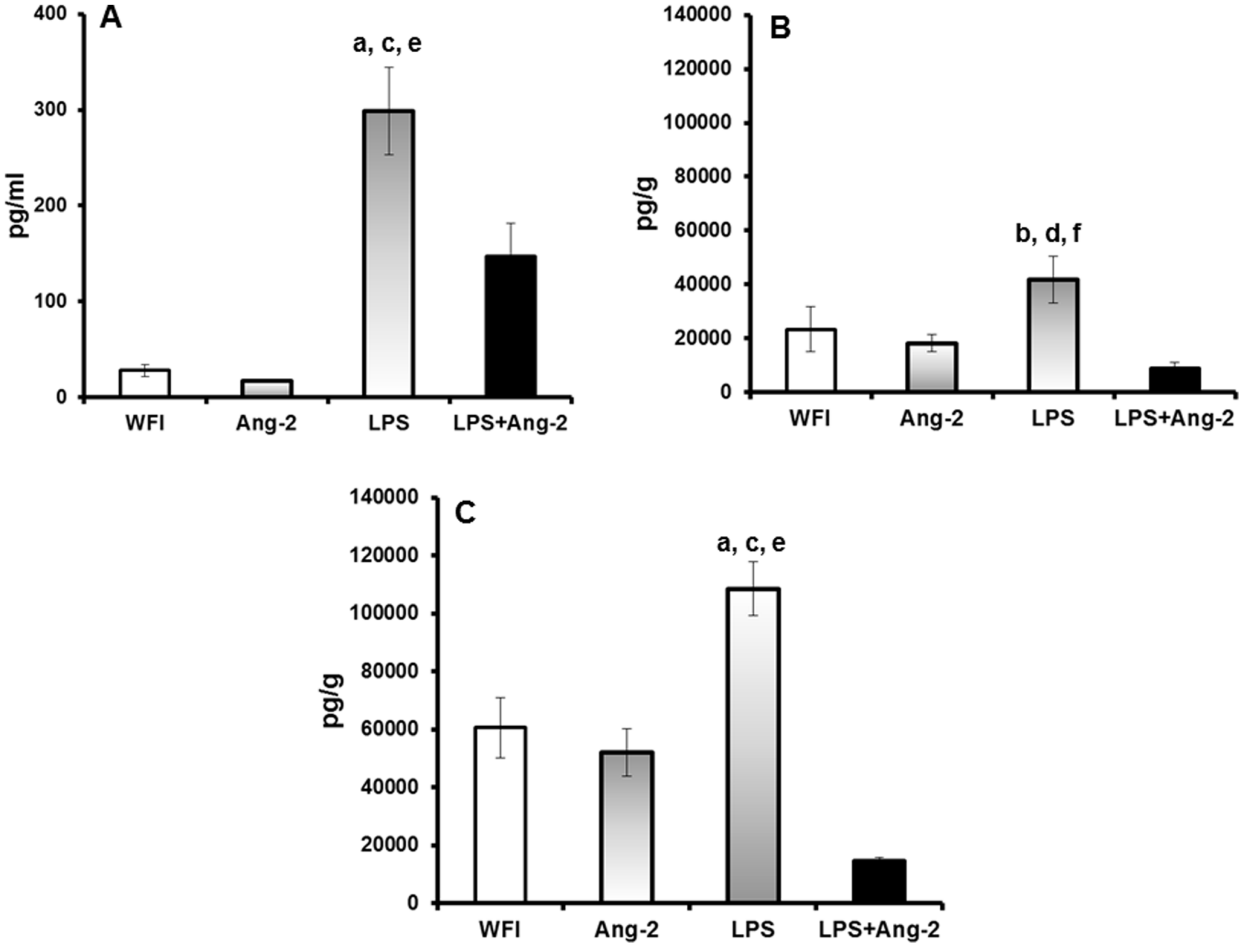
The effect of Ang-2 on TNFα. Pregnant female mice were challenged at 14.5 days after plugging with water for injection (WFI, group A, n = 5), with recombinant human angiopoietin-2 (Ang-2 B, n = 5), with lipopolysaccharide of *Escherichia coli* O55:B5 (LPS, n = 5) and with Ang-2 followed after two hours with LPS (Ang-2+LPS, n = 7). One day after challenge with LPS, mice were sacrificed. Concentrations of TNFα were measured in the sera of mothers (panel A); in the homogenates of fetuses (panel B); and in the homogenates of the placentas (panel C). P values of statistically comparisons between groups: Comparisons LPS vs WFI ^a^p<0.0001 LPS vs WFI; ^b^p: 0.012. Comparisons LPS vs Ang-2 ^c^p<0.0001; ^d^p: 0.006. Comparisons LPS vs LPS+Ang-2 ^e^p<0.0001; ^f^p: 0.006.

Pre-treatment with Ang-2 was accompanied by a considerable anti-inflammatory effect. More precisely, circulating TNFα of mothers and tissue concentrations of TNFα were significantly lower in the fetuses and in the placentas of group D than of group C (Figure 5).

## Discussion

PTD remains an unmet medical need in developed countries and a major cause of morbidity in underdeveloped countries [10]. The presented results indicate that Ang-2 may interfere with the pathogenesis of PTD and accentuate the effects of an infectious process. In women who deliver prematurely, Ang-2 circulates in the maternal circulation at increased levels by the end of first trimester. Our results from the designed animal model suggest that Ang-2 cannot harm the embryo *per se*; this is taking place only after challenge with LPS.

Recent studies of our group have shown that Ang-2 antagonizes the activity of LPS in vitro though an interaction with TLR4 [7]. Survival was prolonged in mice either pre-treated with Ang-2 two hours before challenge with *P.aeruginosa* or treated with Ang-2 30 minutes after challenge with *P.aeruginosa* [6]. When Ang-2 is added in the growth medium of U937 monocytes stimulated by heat-killed microorganisms, monocytes are hampered for further TNFα production. Based on these previous findings for the in vitro effect of Ang-2 on monocytes, we propose the following explanation for PTD in our model: normally LPS induces PTD and stimulates an intense pro-inflammatory response. As a counter-balance, Ang-2 is produced to down-regulate TNFα hyper-production. In this case, fetal angiogenesis is disrupted as evidenced by the decreased fetal concentrations of Evans blue and this leads to PTD.

Angs are involved in the process of placental growth and maturation. Specifically, they participate in the process of endothelial cell migration and increased vascular permeability while attending formation of nascent placental vessels [11]. Ang-1 and Ang-2 are both ligands for the extracellular domain of the tunica internal endothelial cell kinase-2 (Tie2) receptor [4], [5]. Binding of Ang-1 to Tie2 promotes endothelial cell migration and survival. Ang-2 is the endogenous antagonist of Ang-1 and plays an important role in the regulation of vascular remodeling [5].

At present, several studies indicate that Ang-2 is important for normal female reproduction. Early during human pregnancy, Ang-2 is expressed in syncytiotrophoblast and invasive cytotrophoblast [12], while it is suggested that Ang-1/Ang-2 may play an important role in the cross talk between blastocyst and maternal endometrium during the process of embryo implantation [13]. Overexpression of the *Ang-2* gene promotes placental vascular plasticity by remodeling the perivascular extracellular matrix and by increasing vascular luminal area, subsequently promoting interstitial edema whatever increases placental weight [14].

This is not the first study showing elevated circulating Ang-2 early during pregnancy [11]. It is actually suggested that in pregnancies complicated by retardation of intrauterine growth, the increase of Ang-2 is less profound [15]. To this end, measurement of serum Ang-2 at 6–8 weeks of gestation can designate the risk of miscarriage, since levels are significantly decreased in failed pregnancies [16].

Ang-2 is known to increase vascular permeability. As such it would have been expected that the concentration of Evans Blue in the fetus would be extremely high in a state of hyper-Ang-2. Evans blue is a dye which has a very high affinity for serum albumin. It is demonstrated that the dye complexes cannot leave the uterine vessels in areas which have not exhibited the vascular permeability response of that macromolecule [17]. As a consequence, the action of Ang-2 to prime the effect of LPS should be explained in light of two characteristics of the studied kinetics of Evans Blue: the lack of effect on placenta; and the increased concentrations in the maternal tissues. Previous findings of our group in septic mice [6] would suggest that in the current setting Ang-2 aims to antagonize the intrauterine infection and its deleterious effects on the embryos. As a complication, Ang-2 reverses the permeability of the placenta towards the maternal tissues that mandate increased perfusion to combat the infection. This deprives perfusion from the embryo. In parallel, the anti-inflammatory effect of Ang-2 on TNFα decreases embryonic TNFα to levels lower than those of the normal embryos. Taking into consideration the role of TNFα in angiogenesis [18], it is evident that embryos experience further perfusion deprivation. All these events finally lead to PTD.

It may be argued that a model of LPS injection is not the best prototype to study PTD since intrauterine infections account for less than 30% of cases of PTD. However it is the most broadly used model to study PTD because a) many cases of infections ending in PTD run subclinical [19]; and b) intrauterine inflammation occurs in more than 60% of cases of PTD [20]. Increased concentrations of pro-inflammatory cytokines and mainly of TNFα have been found as early as the first 16 to 19 weeks of pregnancy in the amniotic fluid of women who delivered prematurely [19]. TNFα is over-produced in animal models of PTD induced by the injection of LPS or of shiga toxin and it is considered to be a driver for PTD. In these models, pre-treatment with TNFα blockers like etanercept or anti-TNFα antibody prevented PTD and decreased excess concentrations of TNFα in the maternal circulation and in the embryos [20], [21]. It is assumed that TNFα acts by priming over-expression of cycloxogenase-2 and the subsequent production of prostaglandins [21]. In the present study, pre-treatment with Ang-2 decreased TNFα probably implying that the major mechanism of action of Ang-2 leading to PTD is deprivation of embryonic perfusion.

The presented results suggest for an important role of Ang-2 in PTD that is taking place in the field of intrauterine infection. This is mediated through alterations of the vascular permeability of the placentas leading to deprivation of perfusion of the embryos. Results may be promising for the development of therapies aiming at the early prevention of PTD.
